# Intraoperative irradiation in breast cancer: preliminary results in 80 patients as partial breast irradiation or anticipated boost prior to hypo-fractionated whole breast irradiation

**DOI:** 10.1007/s12094-021-02728-0

**Published:** 2021-11-18

**Authors:** X. Li, J. Sanz, N. Argudo, M. Vernet-Tomas, N. Rodríguez, L. Torrent, E. Fernández-Velilla, O. Pera, Y. Huang, P. Nicolau, M. Jiménez, M. Segura, M. Algara

**Affiliations:** 1 Autonomous University of Barcelona, Barcelona, Spain; 2 Pompeu Fabra University, Barcelona, Spain; 3 Radiation Oncology Department, Hospital del Mar, Parc de Salut Mar, C/. Del Gas s/n Edificio B, sótano-2, 08003 Barcelona, Spain; 4 Radiation Oncology Research Group, Institut Municipal d’InvestigacióMédica (IMIM), Barcelona, Spain; 5 Breast Unit, Hospital del Mar, Barcelona, Spain

**Keywords:** Breast cancer, Intraoperative irradiation, Partial breast irradiation, Boost, Hypofractionation, Patient reported outcomes

## Abstract

**Purpose:**

To present the first results of intraoperative irradiation (IORT) in breast cancer with a low-energy photon system used as partial breast irradiation (PBI) or as an anticipated boost before whole breast hypo-fractionated irradiation (IORT + WBI), concerning tolerance, side effects, quality of life, and patient-reported outcomes.

**Materials and methods:**

Eighty patients treated with an Intrabeam^®^ system of 50 kV X-rays received a 20 Gy dose intraoperatively were included. Moderate daily hypofractionation of 2.7 Gy in 15 fractions up to 40.5 Gy was administered if high-risk factors were present. Acute post-operative toxicity, surgery complications, chronic toxicity, patient-reported cosmesis and Breast-Q questionnaire were performed at follow-up visits.

**Results:**

Thirty-one patients were treated as PBI and the remaining 49 as IORT + WBI. Only the IORT + WBI group presented acute toxicity, mainly mild acute dermatitis (11 patients) and one subacute mastitis. A total of 20 patients presented fibrosis (18 patients grade I, 2 patients grade II), 15 (30.5%) patients in the IORT + WBI group and 3 (9.6%) patients in the group of PBI. The cosmesis evaluation in 73 patients resulted poor, fair, good or excellent in 2, 7, 38 and 26 patients, respectively. In PBI group Breast-Q scored higher, especially in terms of their psychosocial well-being (78 vs 65) and satisfaction with radiation-induced toxicity (77 vs 72, respectively) compared to IORT + WBI group.

**Conclusion:**

IORT is a well-tolerated procedure with low toxicity, good cosmesis and favorable patient-reported outcomes mainly when administered as PBI.

## Introduction

Breast cancer has a high incidence, representing 11.6% of all neoplasms in all countries [1]. The results of randomized studies have shown that conservative surgery and radiotherapy are as effective as mastectomy in terms of local control [2, 3], and conservative treatment even can offer better survival rate [4]. Whole-breast irradiation (WBI) reduces ipsilateral recurrence [5], but can produce skin toxicity and fibrosis, especially when boosting the tumor bed [6]. Accelerated partial breast irradiation (APBI) focuses irradiation only to tumor bed with margin, as recurrences occur more frequently in this area [7] and allows shorter treatment duration while sparing healthy tissue. Several randomized trials comparing APBI to WBI demonstrated similar tumor control after 5 years in selected patients [8]. GEC- ESTRO and ASTRO have provided guidelines for the selection of treatment in patients eligible for APBI [9, 10]. Many APBI techniques have been developed, including brachytherapy [11], external radiation therapy [12], and intraoperative radiation therapy (IORT) [13, 14]. IORT allows an extremely short radiation treatment time during surgery and decreases the hospital visits for adjuvant radiation therapy. Also, direct irradiation of the surgical bed is performed and allows better protection of nearby organs at risk. Moreover, intraoperative radiotherapy can administer tumor boost dose and avoid the geographic miss in patients who require WBI [15].

This article presents the first results of the application of IORT with a 50 kV photon system, both as PBI or as an anticipated boost before hypo-fractionated irradiation (IORT + WBI), concerning tolerance, side effects, quality of life, and acceptance reported by patients.

## Materials and methods

### Inclusion of patients

Patients evaluated in our breast unit between June 2018 and February 2020 who received IORT were included in the study and distributed in two groups: the PBI group, that, according to ASTRO consensus [10] for partial breast irradiation, comprised patients with infiltrating tumors smaller than 3 cm, grade I or II, no extensive or high-grade intraductal component, free surgical margins, positive hormone receptors, and uninvolved nodes, or also pure intraductal tumors grade I or II less than 2.5 cm, detected by screening mammography; and the IORT + WBI group, comprising cases that did not meet the criteria for exclusive PBI (including age less of 50 years or receiving neoadjuvant treatment) and for who the IORT was complemented with external radiotherapy after surgery or after adjuvant systemic treatment when indicated.

### IORT procedure

**Fig. 1:**
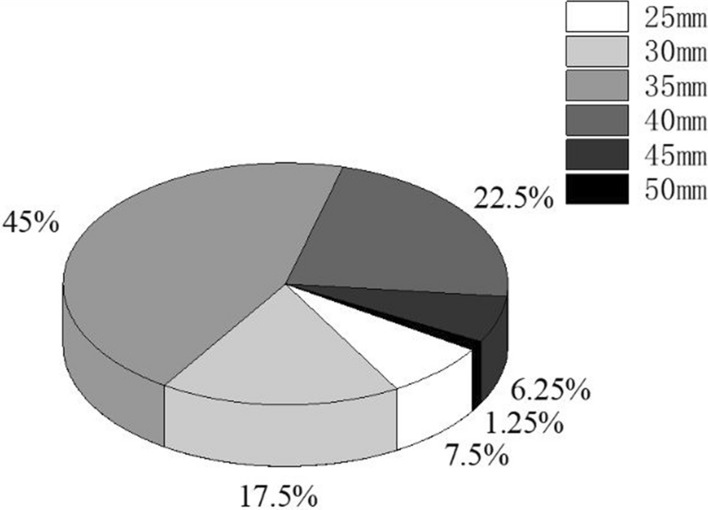
Percentages of different utilized applicators

Patients were given a post-lumpectomy radiation therapy dose before the completion of their surgery using an Intrabeam^®^ system (Carl Zeiss Meditec, Oberkochen, Germany) that utilizes a point source of X-rays of 50 kV energy that provides the dose by means of a spherical applicator that allows effective irradiation at 1–1.5 cm depth. All patients received a 20 Gy dose prescribed at applicator surface. The irradiation time varied depending on the applicator size used and ranged between 17 and 40 min. In Fig. 1 distribution of diameter applicator sized are shown. The chosen applicator was attached to the accelerator and easily inserted into the surgical cavity and held in position by a provisional purse string suture. Irradiation was always carried out with staff remaining outside the operating room. After irradiation, the provisional suture was released, the applicator was withdrawn, and the intervention was completed.

### Postoperative evaluation and treatment

In the post-operative evaluation, all patients were visited 3–4 weeks after surgery to determine if additional irradiation was needed based on the pathological result and our hospital guidelines. When necessary, WBI was scheduled after surgery or after chemotherapy-based systemic treatment. In these cases, the breast was irradiated with moderate daily hypofractionation of 2.7 Gy in 15 fractions up to 40.5 Gy total dose.

### Follow-up

The follow-up was carried out at 1, 3, 6, 9, and 12 months, evaluating acute post-operative toxicity, surgery complications, chronic toxicity, patient-reported cosmesis, and also the health-related quality of life using the Breast-Q questionnaire designed to reflect the patient's perception of the results of surgery and their degree of satisfaction in different aspects related to the treatment of their breast cancer [16]. The questionnaires were answered one year after the surgery, obtaining a score from 0 to 100 in the different domains included, where a higher value represents a more favorable result. Change of more than 10 points on a scale from 0 to 100 is considered clinically relevant results [17].

## Results

### Patient and tumor characteristics

A total of 80 patients with early breast cancer treated with IORT were included. Follow-up was between 12 and 28 months (median = 18). The characteristics of the patients were: mean age of 63 years (range 30–86), 63 patients (78.7%) were postmenopausal, 14 (17.5%) were premenopausal and 3 (3.8%) were perimenopausal.

There was a history of previous neoplasia in 13 patients of which 10 were contralateral and 3 were ipsilateral, thus treated at the time of relapse (one without previously receiving radiotherapy after the first conservative treatment). The clinical and pathological characteristics of the included patients are presented in Table 1. The margin after resection was free in all patients. Five patients (4.3%) were treated with neoadjuvant chemotherapy and 18 patients (22.5%) with adjuvant chemotherapy.

**Table 1:** Characteristics of the patients

Number of patients	Total *n* = 80	PBI *n* = 31	IORT + WBI *n* = 49
Age	63 (30–86)	64 (48–86)	62 (30–84)
Tumor size	12.1 ± 8.45	10.1 ± 4.3	13.4 ± 10.1
Menopause			
Premenopause	14 (17.5%)	4 (12.9%)	10 (20.4%)
Perimenopause	3 (3.8%)	0	3 (6.1%)
Postmenopause	63 (78.7%)	27 (87.1%)	36 (73.5%)
Breast laterality			
Left	40 (50%)	16 (51.6%)	24 (49%)
Right	40 (50%)	15 (48.4%)	25 (51%)
Quadrant location			
Superior-external	45 (56.2%)	19 (61.3%)	26 (53.1%)
Superior-internal	15 (18.7%)	7 (22.6%)	8 (16.3)
Mid-superior	10 (12.5%)	3 (9.7)	7 (14.3%)
Mid-inferior	1 (1.3%)	0	1 (2%)
Medial	9 (11.3%)	2 (6.5%)	7 (14.3%)
Stage			
cT0	7 (8.75%)	3 (9.7%)	4 (8.2%)
cT1	66 (82.5%)	27 (87%)	39 (79.6%)
cT2	5 (6.3%)	0	5 (10.3%)
Relapse	2 (2.5%)	1 (3.3%)	1 (2%)
cN0	73 (91.2%)	30 (96.8%)	43 (87.7%)
cN1	6 (7.5%)	1 (3.2%)	5 (10.3%)
cN2	1 (1.3%)	0	1 (2%)
Histology			
Ductal infiltrating carcinoma	72 (90%)	27 (87.1%)	45 (92%)
Lobular infiltrating carcinoma	1 (1.2%)	1 (3.2%)	0
Ductal carcinoma in situ	7 (8.7%)	4 (12.9%)	5 (6.1%)
Grade			
Grade I	22 (27.5%)	15 (48.4%)	7 (14.3%)
Grade II	46 (57.5%)	15 (48.4%)	31 (63.3%)
Grade III	12 (15%)	1 (3.2%)	11 (22.4%)
Intraductal component			
Yes	48 (60%)	14 (45.2%)	34 (69.4%)
No	32 (40%)	17 (54.8%)	15 (30.6%)
Lymphovascular invasion			
Yes	10 (12.5%)	0	10 (20.4%)
No	70 (87.5%)	31 (100%)	39 (79.6%)
Lymph node involvement			
pN0	69 (86.3%)	30 (96.8%)	39 (79.6%)
pN1	9 (11.3%)	1 (3.2%)	8 (16.3%)
pN2	2 (2.5%)	0	2 (4%)
Ki 67			
< 25	69 (86.3%)	28 (90.3%)	41 (83.7%)
≥ 25	11 (13.7%)	3 (9.7%)	8 (16.3%)
Her2			
Positive	16 (20%)	5 (16.1%)	11 (22.4%)
Negative	57 (71.3%)	23 (74.2%)	34 (69.4%)
Antiestrogenic treatment	69 (86.2%)	28 (90.3%)	41 (83.7%)

### Received treatments

Of the patients included in the study, 31 (38.8%) were treated with intraoperative radiotherapy as PBI, without requiring subsequent adjuvant irradiation. The remaining 49 patients (61.3%) received IORT + WBI and in nine patients (11.2%) the lymph node areas were also irradiated.

### Postoperative and acute radiation toxicity

Postsurgical seroma occurred in 38 cases, 17 (54.8%) patients in the PBI group, and 21 (42.9%) patients in the IORT + WBI group. Three patients required drainage puncture. The toxicity of the patients who received external radiotherapy is presented in Table 2. Only in the IORT + WBI group acute skin toxicity was reported, mainly mild dermatitis in 11 patients, and one patient presented subacute mastitis at mid-term follow-up.

**Table 2:** Toxicity of the patients included in both groups

Toxicity	Total *N* = 80	PBI *N* = 31	IORT + WBI *N* = 49
Seroma			
No	42 (52.5%)	14 (46.2%)	28 (57.1%)
Yes	38 (47.5%)	17 (54.8%)	21 (42.9%)
Duration	226 (20–810)	233 (21–720)	221 (20–810)
Acute toxicity			
Dermatitis			
G1	6 (7.5%)	0	6 (12.2%)
G2	5 (6.3%)	0	5 (10.2%)
Subacute mastitis	1 (1.3%)	0	1 (2%)
Fibrosis			
G0	59 (73.8%)	27 (87.2%)	32 (65.3%)
G1	16 (20%)	3 (9.6%)	13 (26.5%)
G2	2 (2.5%)	0	2 (4%)
Unknown	3 (3.7%)	1 (3.2%)	2 (4%)
Cosmesis			
Excellent	27 (33.8%)	15 (48.4%)	12 (24.5%)
Good	38 (47.5%)	15 (48.4%)	23 (46.9%)
Fair	7 (8.8%)	1 (3.2%)	6 (12.2%)
Bad	2 (2.5%)	0	2 (4.1%)
Unknown	6 (7.5%)	0	6 (12.2%)

### Chronic toxicity and cosmesis

A total of 20 patients presented fibrosis (18 patients with grade I fibrosis, 2 patients with grade II) distributed as follows: 15 (30.5%) patients in the IORT + WBI group and 3 (9.6%) patients in the PBI group. The cosmesis evaluation in 74 patients resulted in 27, 38, 7, and 2 patients with excellent, good, fair, and poor cosmesis, respectively.

### Patient reported outcomes

**Fig. 2:**
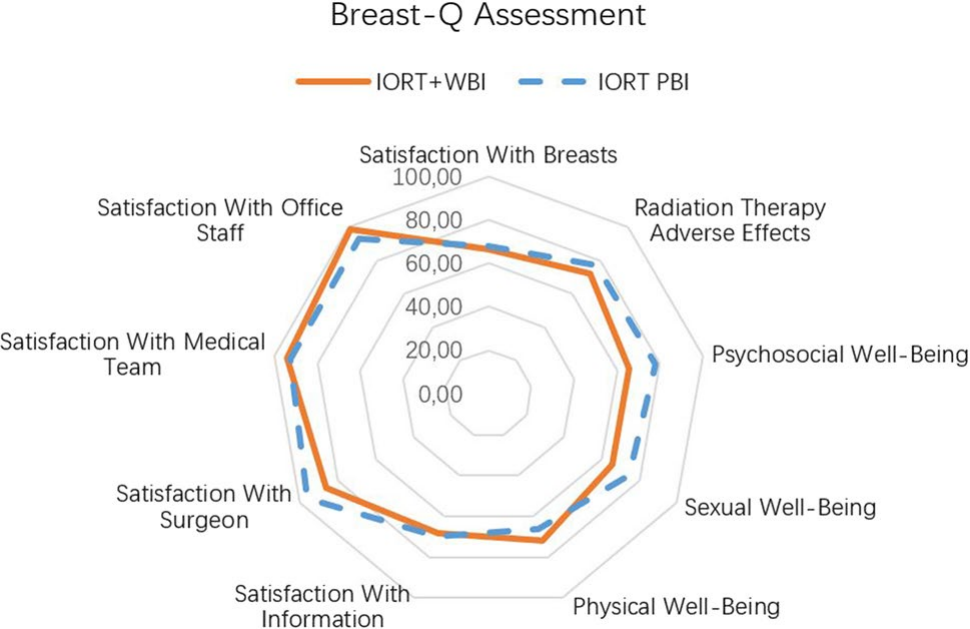
Breast-Q results for whole series and groups of PBI or IORT + WBI

Of the total patients included, 67 patients (84%) completed the Breast-Q questionnaires, 26 (84%) in the PBI group, and 41 (84%) in the IORT + WBI group. It is noteworthy that patients with PBI reported the highest scores in general, especially in terms of their psychosocial well-being (78 vs 66). and were also more satisfied with radiation-induced toxicity (77 vs 72, respectively). In addition, PBI patients reported better sexual well-being and satisfaction with their surgeon than IORT + WBI patients (75 vs 65, 96 vs 86). Satisfaction with breasts and satisfaction with information were seen without significant difference between IORT + WBI group and PBI group (66 vs 68, 68 vs 70). On the other hand, physical well-being, satisfaction with medical team and satisfaction with office staff in IORT + WBI group performed better (72 vs 66, 94 vs 92, 98 vs 93, respectively), see Fig. 2.

## Discussion

### PBI techniques and outcomes

APBI has good efficacy in selected patients as shown in several trials. In the Pòlgar study utilizing brachytherapy, were found no differences in local recurrence between WBI and APBI (5.1% vs 5.9%). The APBI patients had significantly fewer moderate to severe late side effects, and excellent cosmetic result [18]. APBI can also be performed by external irradiation [19]. In the RAPID Trial [20], APBI was not inferior to WBI in preventing ipsilateral relapse in node-negative breast cancer. The NSABP B-39/RTOG 0413 trial has confirmed this equivalence and the absolute difference at 10 years was also small (< 1.6%) between arms [21].

### IORT rationale and trials

Another alternative to partial irradiation is IORT, a suitable technique which allows small volumes of treatment and skin protection with a positive impact on the late toxicity and cosmesis. In the updated ASTRO consensus and after revision of the published evidence, IORT was included as an option to perform PBI, remarking the need of careful selection of patients to be included outside a clinical trial (“suitable group”) [10]. The radiobiological differences of the available IORT available techniques made difficult their direct comparison.

The first experiences of IORT have been carried out with mobile accelerators that provide irradiation by means of an electron beam. The ELIOT trial included 1305 patients, comparing WBI to PBI with electron IORT. At five years follow-up, the relapse rate was significantly higher in IORT group than in WBI group, and overall survival was not different between the two groups [22]. The improved patient selection allows better results in terms of disease control [23].

More recently, the TARGIT-A trial, using 50 kV X-rays with Intrabeam^®^, 3451 patients were randomized to receive WBI (1730 patients) or IORT (1721 patients). Wound-related complications were the same between groups, but there was significantly less grade 3 or 4 toxicity related to radiotherapy complications with IORT than with WBI [24]. Also, a better cosmesis result is observed through computer-assisted objective system [25], and overall better quality of life [26]. With a longer follow-up (median 8.6 years, maximum 18.9 years), no statistically significant differences were found for local recurrence-free survival (hazard ratio 1.13; 95% CI 0.91–1.41; *p* = 0.28) [27], but there were significantly fewer deaths in the IORT group than in the WBI group, attributable to fewer deaths from cardiovascular causes and other cancers.

In our study patients submitted to intraoperative PBI resulted in an excellent tolerance and mild toxicity. The main secondary effect was seroma presented in half of the series but in the majority of cases was resolved without treatment. Only 9.6% of patients present mild fibrosis and the cosmetic results is good or excellent in 96% of cases.

### IORT + WBI

In addition, IORT is also an option for performing an anticipated tumor bed boost [28]. Fastner et al. [29] published the results of IORT with electrons as a boost and showed that reduce recurrence in breast cancer, with only 16 (0.8%) recurrences observed in the breast (*p* = 0.031). A randomized case III trial comparing 10 Gy IORT as a boost with WBI and external irradiation boost at standard fractionation demonstrated iso-efficacy of both arms while obtaining better cosmetic results in IORT boost arm [30]. In HIOB trial combining electron IORT and hypo-fractionated WBI the tolerance was excellent and cosmesis appearance was not altered after 3 years evaluation [31]. Our results in the group of IORT + WBI have shown acute dermatitis after hypo-fractionated WBI and a discrete trend to higher fibrosis and less favorable cosmesis but comparable to standard fractionation schedules after IORT.

### Patient-reported outcomes

There is little research about patient-reported outcomes in the setting of IORT. Patients in TARGIT A trial tended to self-report better outcomes for breast-related quality of life (QOL), and they experienced fewer symptoms and better results in breast-related QOL [32]. In our series, the PBI patients have overall higher scores in the quality of life and satisfaction tests, recover their psychosocial well-being earlier, and have a lower perception of radiotherapy adverse effects. However, they show poorer scores on the physical well-being scale, which could be partially explained by the slightly older age of patients selected for PBI. In terms of patient comfort, IORT prolongs the surgical procedure for only an additional short period but, dramatically shortens, or in selected cases perhaps even replaces, post-operative radiation therapy. In patients with IORT + WBI it seems to have no impact of hypo-fractionated irradiation at well-being evaluations.

## Conclusions

According to our study, IORT shows low toxicity, good cosmesis, and good quality of life for patients. When administered as an anticipated boost, prior to hypo-fractionated WBI there is a trend towards greater toxicity, but the cosmesis results remain quite good and comparable to those reported in the literature.

## Data Availability

All data are available for analysis and review in electronic records.
